# Contemporary Review of Borderline Resectable Pancreatic Ductal Adenocarcinoma

**DOI:** 10.3390/jcm8081205

**Published:** 2019-08-12

**Authors:** Morgan Bonds, Flavio G. Rocha

**Affiliations:** Section of General, Thoracic and Vascular Surgery, Virginia Mason Medical Center, Seattle, WA 98101, USA

**Keywords:** borderline resectable, pancreatic cancer, neoadjuvant therapy

## Abstract

Borderline resectable pancreatic adenocarcinoma (PDAC) presents challenges in definition and treatment. Many different definitions exist for this disease. Some are based on anatomy alone, while others include factors such as disease biology and patient performance status. Regardless of definition, evidence suggests that borderline resectable PDAC is a systemic disease at the time of diagnosis. There is high-level evidence to support the use of neoadjuvant systemic therapy in these cases. Evidence to support the use of radiation therapy is ongoing. There are ongoing trials investigating the available neoadjuvant therapies for borderline resectable PDAC that may provide clarity in the future.

## 1. Introduction

Pancreatic ductal adenocarcinoma (PDAC) is an aggressive malignancy. While it is the 14th most common cancer worldwide, PDAC is the seventh leading cause of cancer deaths [1]. The high rate of mortality from this disease is attributed to the advanced stage at diagnosis. Surgical resection offers the only potential for cure from PDAC. However, only 20–30% of PDAC patients will undergo resection, either due to locally advanced or metastatic disease.

The treatment of PDAC continues to change as clinicians and investigators discover more about the biology of the disease. Historically, a pancreatic mass that could be resected upfront would undergo surgery. Systemic adjuvant therapy would then be considered based on the surgical pathology. A correlation of R0 resection and node negative pathology with improved survival was demonstrated in patients who underwent resection [2,3,4]. This finding led to pancreatic specialists attempting to determine which PDAC patients would benefit from neoadjuvant therapy prior to resection, in order to increase the rate of R0 margins and decrease node positivity [5,6]. This consideration fostered the preoperative categorization of PDAC into resectable, borderline resectable, locally advanced, and metastatic disease, particularly for investigative purposes.

The aim of this article is to review borderline resectable pancreatic cancer. It will define borderline resectable disease and describe how to differentiate it from locally advanced PDAC. Additionally, this article will address the evolution of PDAC therapy to current recommendations. We will also discuss ongoing clinical trials hoping to advance the current knowledge and treatment of patients with borderline PDAC.

## 2. Definition of Borderline Resectable Disease

The definition of resectable PDAC has evolved over the past two decades, and as resection is the sole opportunity for cure, this definition is critical. Most definitions for borderline resectable PDACs include tumors that are not clearly resectable due to some involvement of the mesenteric vessels [5,6,7,8]. However, over the years, investigators have used different definitions for borderline resectable PDACs and, occasionally, use the terms borderline resectable and locally advanced interchangeably. This ambiguity complicates the interpretation of study results, including the survival rates associated with alternative treatment pathways.

As the majority of borderline resectable PDAC definitions use anatomic determinations, a short discussion regarding pancreatic imaging is warranted. Multidetector computed tomography (CT) of the abdomen with pancreatic protocol, comprised of an arterial and venous phase with fine cuts through the gland, is the recommended imaging modality of choice [9]. This allows visualization of the pancreatic mass and its relation to the portal vein (PV), superior mesenteric vein (SMV), superior mesenteric artery (SMA), and celiac axis (CA), in three planes. An alternative to preoperative staging for resectability to abdominal CT with IV contrast is magnetic resonance imaging (MRI), however this has been limited to institutions with expertise. Endoscopic ultrasounds (EUS) can be helpful in defining the local T and N stage of the tumor, but lack the ability to completely view the liver and peritoneum, missing common areas of metastases. The individual contributions as well as the combination of CT and EUS have also been examined in regards to their discriminative ability to predict vascular involvement, particularly in the setting of neoadjuvant therapy. Both modalities have higher sensitivity and low specificity for identifying venous invasion and close resection margins (<1 mm) in patients who have received neoadjuvant therapy compared to those undergoing upfront resection [10]. These findings indicate that EUS is not routinely recommended in addition to CT for the evaluation of resectability, particularly after neoadjuvant therapy.

**Figure 1:**
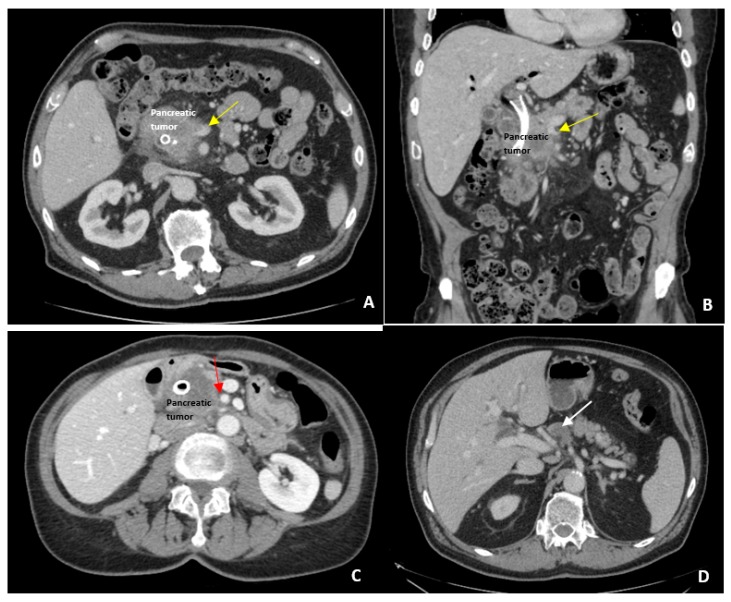
Borderline Resectable Pancreatic Cancer Imaging. Examples of borderline resectable pancreatic adenocarcinoma. (**A**) Axial view of pancreatic tumor narrowing the superior mesenteric vein (SMV) indicated by the yellow arrow; (**B**) The same pancreatic mass narrowing the SMV on coronal view; (**C**) Abutment (<180 degrees) of the superior mesenteric artery (SMA) by pancreatic head tumor indicated by the red arrow; (**D**) Large regional lymph node (white arrow) that was later biopsy proven to be metastatic pancreatic adenocarcinoma.

The concept of borderline resectable PDAC was first suggested by Mehta et al. in 2001 [5]. This group used the term “marginally resectable”, which was defined as 180 degree or more involvement of the PV, SMV, or SMA for the length of 1 cm or more [5]. Interestingly, this study used poor performance status as exclusion criteria but not as part of their definition for resectability. Five years later, the MD Anderson group published an expanded definition of borderline resectable PDAC. They defined tumor abutment of 180 degrees or less on the SMA, short segment encasement or abutment of the common hepatic artery (typically at the gastroduodenal artery origin), and short segment occlusion of the SMV/PV with normal vein above and below to allow reconstruction as borderline resectable lesions [6] (Figure 1).

A consensus statement from the Americas Hepato-Pancreato-Biliary Association (AHPBA), the Society of Surgical Oncology (SSO), and the Society for Surgery of the Alimentary Tract (SSAT) provided another definition for borderline resectable PDAC. Like previous definitions, it focuses on anatomical criteria. Per this consensus statement, a tumor is borderline resectable if there is abutment of the SMV or PV with or without deformity, encasement of the SMV or PV without arterial involvement, or short segment occlusion that allows safe resection and reconstruction without evidence of distant metastases. Additionally, the gastroduodenal artery can be encased with short segment encasement or abutment of the hepatic artery without involving the celiac access or abutting the SMA ≤ 180 degrees in circumference and be defined as borderline resectable [9].

The major critique of these definitions is their subjective nature. What one surgeon may deem a reconstructable vein may be inoperable to another surgeon. Additionally, there is no consideration to biological factors or the performance status of the patient. The MD Anderson group expanded their definition of borderline resectable PDAC in a 2008 article that addressed cancer biology and patient well-being; in this paper, the anatomical definition was unchanged and constituted one group of borderline resectable patients. Two other groups were also defined as borderline resectable, including patients with suspicious, non-diagnostic CT evidence of extrapancreatic metastatic disease or confirmed lymph node (N1) metastases, as well as patients with poor performance status or severe comorbidities [11].

The International Association of Pancreatology (IAP) developed criteria for borderline resectable PDAC in an attempt to further address the role of biology, patient performance status, and the subjective language of other definitions. In this statement, borderline SMV/PV involvement is defined as 180 degree tumor contact or occlusion that does not exceed the inferior border of the duodenum, and SMA tumor contact less than 180 degrees without stenosis or deformity. The common hepatic artery is considered a borderline resectable disease if the proper hepatic artery and celiac artery are not involved. By eliminating the vague language about reconstruction, this anatomical definition of borderline resectability can be used in all pancreatic surgery practices. The IAP consensus group further laid out a biological definition of borderline resectable PDAC. These patients appear to have an anatomically resectable disease but have the potential for distant metastases, including a CA 19-9 greater than 500 units/mL or lymph node disease proven via biopsy or positron emission tomography (PET) scan. They conclude with a conditional borderline definition that includes patients with anatomically resectable tumors and an Eastern Cooperative Oncology Group (ECOG) performance status of 2 or greater [8].

No single definition of borderline resectable PDAC has been agreed upon and made universal. The intergroup consensus definition used in the Alliance 021101 has been accepted by the majority of PDAC clinicians and for the design of future clinical trials [12]. Further standardization is necessary to improve our understanding of this disease stage. The definitions of borderline resectable PDAC can be found in Table 1.

## 3. Current Treatment for Borderline Resectable Disease

The current guidelines for the treatment of borderline resectable PDAC tend to be as vague as the definition for this disease. The National Comprehensive Cancer Network (NCCN) guidelines state that a tumor biopsy should be performed, followed by the administration of neoadjuvant therapy prior to resection for this stage of disease [13].

The decision whether to pursue neoadjuvant therapy or surgical resection first has historically been challenging in borderline resectable PDAC. One determinant is the comfort of the surgeon performing the vascular resection and reconstruction. Biological factors also select for management strategy. Elevated preoperative CA 19-9 levels have been shown to be associated with positive nodal metastases, microscopically positive margins, shorter overall survival, and shorter disease-free survival in patients diagnosed with Stage I or II PDAC [14]. This is supported by a review of the National Cancer Data Base (NCDB), which found that elevated CA 19-9 at diagnosis is associated with a decrease in survival when controlled for stage. They concluded that patients with elevated pretreatment CA 19-9 should undergo neoadjuvant chemotherapy, as they could be considered “biologically” borderline resectable [15].

The current guidelines have been updated to reflect the evidence we will present in the remainder of this article. This evidence supports the trend of the chemotherapy first approach, as it is becoming the standard of care.

### 3.1. Neoadjuvant Therapy

The arguments for neoadjuvant systemic therapy in PDAC are numerous. The administration of chemotherapy prior to surgery allows for full dose systemic therapy to be delivered without the delay that can often occur after complex pancreatic resections. Additionally, this strategy allows for the identification of patients with a more aggressive disease, whose cancer may progress while on therapy. The counterargument to neoadjuvant therapy is that a number of patients will never make it to resection, eliminating their only chance for cure.

There is evidence to support the use of neoadjuvant therapy. Pancreatic cancer cells can enter circulation before a histologic mass can be seen in the pancreas. Additionally, it has been shown that these circulating tumor cells can result in distant metastases [16]. This data suggests that PDAC is a systemic disease at the time of diagnosis and necessitates systemic therapy. Treating PDAC systemically first appears to improve oncologic outcomes. A large database study comparing patients undergoing upfront resection to patients receiving neoadjuvant chemotherapy demonstrated a longer survival on multivariable analysis in the neoadjuvant group [17]. Other studies have shown that the poor survival associated with an elevated CA 19-9 at diagnosis can be mitigated by neoadjuvant systemic therapy [15,18]. As a result, interest has grown in studying systemic therapy for all stages of PDAC.

The oncological benefits of neoadjuvant therapy in borderline resectable PDAC was suggested in a 2018 randomized controlled trial in Korea. Patients were randomized to either neoadjuvant chemoradiation using gemcitabine or upfront surgical resection with adjuvant chemotherapy. All but one participant completed neoadjuvant therapy, whereas five of the surgery first cohort did not undergo adjuvant therapy. There was no difference in the number of patients found to be unresectable at the time of exploration between the two groups. Negative resection margins were achieved in 82.4% of the resected neoadjuvant cohort, while only 33.3% of the surgery first cohort had R0 resections and 16.7% had R2 resections. Intention-to-treat analysis found two-year survival to be significantly improved in patients receiving neoadjuvant chemoradiation. The study was terminated early due to the improved oncological outcomes of patients receiving neoadjuvant therapy [19]. The data from this trial support the notion that patients are less likely to receive systemic therapy in the adjuvant setting, a scenario that is not ideal in a disease that is systemic at diagnosis. To date, this is the only completed and published randomized controlled trial that supports the oncological and survival benefits neoadjuvant therapy on borderline resectable PDAC patients.

There is little evidence to support which regimen should be used in the neoadjuvant setting. This is reflected in the NCCN guidelines. At our institution, a gemcitabine/taxane doublet neoadjuvant regimen was associated with increased early survival and comparable perioperative complications in a borderline resectable PDAC cohort, although the majority of the patients in this study received additional adjuvant therapy, including postoperative radiation [20]. Another study specifically looking at borderline PDAC patients with arterial involvement showed that R0 resection was higher in those patients who received gemcitabine plus S-1 neoadjuvant therapy compared to those who had resection as the initial treatment. Additionally, median overall survival was higher in patients who underwent neoadjuvant gemcitabine plus S-1 than those who did not (27.1 months versus 11.6 months) [21]. Although gemcitabine has historically been the basis for treating pancreatic cancer, there has been a growing interest in the FOLFIRINOX regimen. This treatment consists of 5-FU, oxaliplatin, and irinotecan. Ferrone et al. published their experience with neoadjuvant FOLFIRINOX in a cohort of borderline resectable and locally advanced PDAC. They found that patients receiving FOLFIRINOX were less likely to have positive lymph nodes, perineural invasion, and lymphovascular invasion compared to resectable patients who went directly to the operating room. There were four patients with only microscopic disease remaining, as well as two complete pathological responses in the neoadjuvant FOLFIRINOX group. These improved pathologic responses resulted in improved disease-free and overall survival in the neoadjuvant group [22]. This study was supported by a larger neoadjuvant FOLFIRINOX cohort, again showing more favorable pathological features and improved survival [23]. Studies comparing neoadjuvant regimens are needed to determine which is the most effective. Table 2 summarizes the outcomes of neoadjuvant systemic therapy from previous studies on borderline resectable PDAC.

### 3.2. Radiation Therapy

The objective of radiation therapy in borderline resectable and locally advanced PDAC is to improve the margin status at resection by destroying the tumor near the abdominal vasculature. Typically, systemic therapy is given in conjunction with locoregional radiation therapy in order to sensitize the tumor to radiation effect.

The routine use of radiation therapy for borderline resectable PDAC is controversial. Surgeons initially wondered if the addition of radiation provides a benefit that outweighs the perceived challenge of operating in a radiated field. Two recent studies using the NCDB database, one of which was propensity matched, suggested that PDAC patients undergoing neoadjuvant chemoradiation had lower overall survival when compared to patients who had neoadjuvant chemotherapy alone [24,25]. Conversely, a single institution study described results using a standardized chemoradiation regimen; patients receiving chemoradiation were compared to patients receiving chemotherapy only. They reported lower rates of node positivity, R1 resection, perineural invasion, and lymphovascular invasion, and local recurrence in the chemoradiation group, and did not observe any statistically significant difference in overall survival between the two treatments [26].

Stereotactic body radiation therapy (SBRT) is a relatively new radiation modality. It is an appealing alternative to external beam radiation due to the limited irradiation of neighboring organs and shorter course of administration. Conversely, this decreased treatment field will lead to the inadequate treatment of peripancreatic lymph nodes. Chuong et al. reported their experience with neoadjuvant SBRT in borderline resectable PDAC. They saw radiographic regression of the tumor in 77% of patients, although only 72% of these patients were able to have the tumor resected at exploration. The vast majority (96.9%) of resected patients had an R0 resection and 9.3% had a complete pathological response. Only 18.8% of the resected borderline tumors required vascular resection or repair [27]. A long-term follow up from the same institution found a median overall survival of 19.2 months and event free survival of 11.9 months for borderline resectable patients undergoing SBRT; when locally advanced and borderline PDAC patients underwent resection after SBRT, the median overall survival increased to 34.2 months [28]. Future study in SBRT is focusing on ways to increase the effect of this modality without dose escalation by using radiosensitizers like nelfinavir [29].

### 3.3. Assessing Response to Neoadjuvant Therapy

Response to neoadjuvant systemic therapy can be difficult to determine. The Response Evaluation Criteria in Solid Tumor (RECIST) methodology has standardized the radiological response of solid tumors to therapy. However, many times PDACs appear unchanged post-treatment on conventional cross-sectional imaging, even in tumors that have had a pathologic response. A 2012 study found that <1% of patients demonstrated regression of tumors from the mesenteric vessels and could not be downstaged to resectable from borderline resectable disease using pancreatic protocol CT. Moreover, only 12% of borderline resectable PDAC patients had a partial response and 0% had a complete response using RECIST criteria. Despite these findings, 80% were able to undergo an R0 resection [30]. This suggests that CT is not the best modality to assess neoadjuvant therapy tumor response. Early evidence suggests that using FOLFIRINOX in the neoadjuvant setting may improve the detection of response using pancreatic protocol CT, but further study is needed to confirm this finding [21].

Other tools can be used to predict the response of PDAC to neoadjuvant therapy. One such tool is the biomarker CA 19-9. As discussed earlier, elevated pretreatment CA 19-9 is evidence of a biologically aggressive disease, but neoadjuvant systemic therapy can mitigate this increased risk. The response of CA 19-9 to neoadjuvant therapy correlates with the tumor response to therapy. One study, using the IMPALA trial cohort, reported that combining a 30% or more decrease in CA 19-9 with the RECIST imaging response improved the detection of patients who would be resectable, based on findings at exploration. The CA 19-9 response was particularly useful in those patients with a RECIST stable disease [31].

Combining CT imaging with functional imaging can improve the detection of tumor response to therapy in borderline PDAC. Flourine-18 fluorodeoxyglucose positron emission tomography (FDG-PET) scans detect glucose uptake within tissue, a marker of tissue metabolism. This is useful in detecting PDAC response to therapy, as live tumor is converted to fibrous scar with minimal metabolic activity. In a 2017 study of resectable and borderline PDAC patients, it was determined that the difference in pancreatic tumor max standardize uptake value (SUV) between pre- and post-treatment FDG-PET images was a better predictor of pathological response than change in tumor size (known as regression index). A regression index of >50% was found to be associated with good pathologic response and was associated with an improved five-year survival (56% versus 36%). Multivariate analysis found that pathological nodal status, resectability, and regression index were significant factors in prognosis [32]. Functional imaging can be used in determining whether a change in neoadjuvant therapy is warranted, in addition to determining resectability.

### 3.4. Surgical Resection

**Figure 2:**
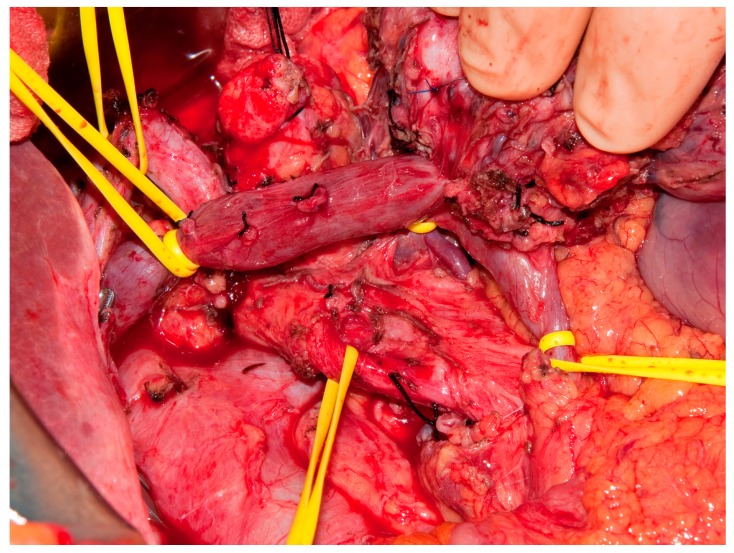
Portal vein confluence involvement of borderline resectable PDAC at time of surgery. A pancreatic ductal adenocarcinoma tumor adhered to the portal vein. Vessel loops have been used to control the venous tributaries prior to resecting the portal vein wall with the specimen. The uncinate process has been resected off the superior mesenteric artery (SMA) in preparation for vein resection.

Patients with anatomically borderline resectable PDAC present a challenge for surgical resection. The involvement of the mesenteric vessels can, at times, require complicated vascular reconstructions in order to achieve an oncologically appropriate resection of the cancer. As discussed above, neoadjuvant systemic and local therapies can reduce the number of borderline resectable PDAC patients that require vascular resection and reconstruction. However, many times these patients will have continued involvement of these structures, both radiographically and in the operating room (Figure 2).

It was initially thought that involvement of the SMV and PV was a poor prognostic factor and indicative of a biologically aggressive disease. This was refuted when studies compared patients undergoing pancreaticoduodenectomy with and without venous resection. There was no difference in pathologic features or tumor DNA index and proliferation fraction; the two groups had an equivalent of two-year survival. This group concluded that vein involvement could simply be attributed to tumor location, not aggressive biology [33]. A larger follow up study reiterated these findings. Patients undergoing venous reconstruction had significantly longer operating times and higher intraoperative blood loss, but multivariate analysis did not show that the procedure had any effect on survival. In fact, the survival of patients undergoing venous resection for PDAC approached that of patients undergoing standard resection, and it was significantly better than the survival of patients with PDAC deemed unresectable due to the involvement of the mesenteric veins [34]. A later European study determined that the type of vascular resection and/or reconstruction had no impact on survival or the disease-free interval. These outcomes were influenced by oncological variables, such as tumor location, tumor stage, and neoadjuvant therapy [35].

Vascular resection and reconstruction are necessary tools in the treatment of borderline resectable PDAC. However, the risks associated with adding a vascular procedure onto a complicated pancreatic resection should not be minimized. One retrospective study with long follow-up found the length of operation and intraoperative blood loss to be significantly higher in groups undergoing both venous and arterial resections during pancreatectomy, although this did not translate to an increase in blood transfusion or major complications [36]. However, a more recent study using the Nationwide Inpatient Sample database looked at 10,206 pancreatectomies, of which 4% had undergone vascular reconstruction. In this sample, there was an increase in major complications compared to patients undergoing pancreatectomy alone, although there was no difference in mortality. This trend was found to persist when analyzing the highest volume centers [37].

Vascular resection in the setting of pancreatectomy may be associated with higher morbidity than pancreatectomy alone. However, evidence suggests it offers patients a survival benefit approaching that of the upfront resectable cohorts, and this approach is the only opportunity some may have for cure. Vascular resection and reconstruction should be planned well in advance to avoid inadvertent mesenteric vascular injuries and prevent additional morbidity. The availability of appropriate consultants (vascular surgeon or other hepato-pancreato-biliary surgeon) should be confirmed prior to scheduling the case. Preparing for vascular resection requires ready access to a vascular graft, either autogenous or synthetic. Intraoperatively, the specimen is ideally prepared by approaching the SMA first, so the venous involvement is the last step before specimen removal and vascular control should be obtained before approaching the involved vessel [38]. It should be noted that the SMA first approach is not always feasible, and the experienced surgeon should be prepared to undertake alternative strategies. These steps will allow for the successful resection of borderline resectable PDAC tumors that involve the mesenteric vasculature. Vascular resection and reconstruction should be performed in patients with borderline resectable PDAC deemed fit to tolerate the additional stress after neoadjuvant therapy has been administered.

## 4. Current Investigation in the Treatment of Borderline Resectable Disease

Obtaining high-quality data on how to best treat patients presenting with borderline resectable PDAC has gained great interest in recent years. At present, several randomized trials investigating different neoadjuvant modalities for resectable and borderline resectable PDAC are underway. This section will briefly discuss these trials and preliminary results where they are available.

A multi-center trial in the Netherlands (PREOPANC-1) is comparing outcomes for patients with upfront and borderline resectable PDAC who received neoadjuvant chemoradiation versus those having upfront resection. Like the Korean study, the preliminary results show a longer overall survival, disease free survival, metastases free interval, and locoregional disease free interval in those undergoing preoperative therapy. Resection rates were not statistically significant between the two groups [39]. Final data from this trial will hopefully support the recommendation that all borderline resectable pancreatic tumors should receive neoadjuvant therapy prior to resection.

Another ongoing clinical trial is the Southwest Oncology Group (SWOG) S1505 trial. This study has randomized patients with upfront resectable PDAC to either FOLFIRINOX or gemcitabine/nab-paclitaxel in the perioperative setting. To date, the study has shown the safety of preoperative chemotherapy with 83% of the total cohort completing the neoadjuvant portion. Exploration was undertaken in 77% of subjects and 73% were able to be resected. Survival data is currently being collected [40]. These results are promising, with a large percentage of participants being able to undergo surgical resection. Although this study is focusing on a different stage of disease, the SWOG S1505 trial may provide valuable evidence regarding which neoadjuvant therapy delivers superior oncological and survival results for all PDAC stages, including borderline resectable.

In Japan, there is a single arm Phase II trial studying the effect of neoadjuvant S-1 oral systemic therapy in combination with radiotherapy in borderline resectable PDAC. Preliminary results were recently reported. Neoadjuvant therapy was completed in 96% of patients. An R0 resection was achieved in 63% of patients with borderline resectable lesions, and 32% of resected tumors were found to have destruction of more than half of the malignant cells. Two-year overall survival was 51% and median progression-free survival was 6.7 months [41]. Another study out of Japan investigated randomized patients with resectable PDAC to neoadjuvant gemcitabine and S-1 chemotherapy versus upfront resection. Both arms received six months of S-1 systemic therapy after resection. The R0 resection rate and morbidity after surgery were not statistically different between the two groups. However, the median overall survival was longer in the neoadjuvant cohort (36.7 months versus 26.6 months), and this difference was statistically significant [42].

The Alliance A021101 trial is investigating whether adding chemoradiation to a neoadjuvant FOLFIRINOX regimen improves outcomes in borderline resectable PDAC. The feasibility trial was completed several years ago; it demonstrated that the FOLFIRINOX followed by capecitabine-based chemoradiation therapy had manageable toxicity, as no patients withdrew due to toxicity. Some patients did experience treatment delays. From this cohort, 68% were able to undergo pancreatectomy. A majority (74%) did require vascular resection and reconstruction. The median overall survival was 22 months. Subgroup analysis found better median survival in patients who underwent resection and patients found to have <5% viable cancer in the pathology specimen [43]. Currently, a randomized controlled trial is underway comparing borderline resectable PDAC patients who received eight cycles of FOLFIRINOX versus patients who received seven cycles of FOLFIRINOX followed by SBRT. Those without disease progression will undergo pancreatectomy followed by four more cycles of FOLFOX6. The primary outcome is 18-month overall survival. The secondary outcomes are margin status and event free survival [44]. This study will provide insight on whether the addition of SBRT improves outcomes seen with neoadjuvant FOLFIRINOX for PDAC.

Finally, we have a safety and feasibility study ongoing at our institution for the neoadjuvant use of gemcitabine/nab-paclitaxel alternating with a 5FU, leucovorin, and liposomal irinotecan (NAPOLI) regimen in resectable and borderline resectable PDAC. Liposomal irinotecan improves drug delivery and the pharmacokinetics of the drug. In this protocol, borderline resectable patients receive alternating 28-day courses of gemcitabine/nab-paclitaxel and NAPOLI for a total of six cycles prior to being assessed for resection. Enrollment is ongoing in this study and we expect preliminary results in 2020.

## 5. Conclusions

To conclude, borderline resectable PDAC is a unique diagnosis, as there are currently no standards on the ideal treatment. Most evidence suggests these patients benefit from at least systemic neoadjuvant therapy, as PDAC is an aggressive, systemic disease at the time of diagnosis. This is especially true for patients considered to be biologically borderline resectable. There may be an additional benefit from local radiation therapy in patients with anatomically borderline resectable disease, however more data is needed in this area. Promising randomized controlled clinical trials are in progress that may provide insight on the best neoadjuvant regimens to use in this disease.

## Figures and Tables

**Table 1:** Definitions of borderline resectable pancreatic ductal adenocarcinoma (PDAC).

	NCCN Definition	AHPBA/SSO/SSAT Consensus Definition	MD Anderson Modified Definition	IAP Consensus Definition
Venous Involvement	Involvement of SMV or PV that distorts, narrows, or occludes the vein with suitable vessel proximal and distal allowing resection and replacement	Involvement of the SMV or PV with or without narrowing, or encasement of the SMV or PV without encasement of nearby arteries, or short segment occlusion from tumor encasement or thrombus allowing resection and reconstruction	Short segment occlusion of SMV, PV, or SMV-PV confluence amenable to vascular resection and reconstruction	Tumor contact of 180 degrees or more circumference or occlusion of the SMV, PV, or SMV-PV confluence that does not exceed the inferior border of the duodenum
Arterial Involvement	Gastroduodenal involvement up to the hepatic artery with short segment encasement or direct abutment of the hepatic artery without extension to the celiac access	Gastroduodenal artery encasement up to hepatic artery with short segment encasement or abutment of the hepatic artery without extension to the celiac access	180 degree or less circumference involvement of the SMA or celiac access or short segment abutment/encasement of the hepatic artery (typically origin of gastroduodenal artery)	Tumor contact of 180 degrees or less circumference of the SMA or celiac access without deformity or tumor contact of the common hepatic artery without abutting the proper hepatic artery or celiac access
Biological	None	None	Concern for extrapancreatic disease (suspicious but non-diagnostic metastatic lesions or locoregional lymph node involvement)	Anatomically resectable PDAC suspicious for extrapancreatic disease (CA 19-9 of 500 units/mL or more or regional lymph node metastases on biopsy or PET-CT)
Performance Status	None	None	Poor performance status (ECOG 3 or more) or significant medical comorbidities that preclude immediate surgery	Anatomically resectable PDAC with poor performance status (ECOG 2 or more)

NCCN—National Comprehensive Cancer Network; PV—portal vein, SMV—superior mesenteric vein; AHPBA—Americas Hepato-Pancreato-Biliary Association; SSO—Society of Surgical Oncology; SSAT—Society for Surgery of the Alimentary Tract; IAP—International Association of Pancreatology; SMA—superior mesenteric artery; ECOG—Eastern Cooperative Oncology Group; PDAC—Pancreatic ductal adenocarcinoma; CA—celiac axis; PET-CT—positron emission tomography-computed tomography.

**Table 2:** Surgical and Pathologic Outcomes after Neoadjuvant Systemic Therapy for Borderline Resectable PDAC by Study.

Study	Year	Pts	Status	Chemo	Resected (%)	Vein Resection (%)	Median Survival (months) All/R/UR	R0 (%)
Mehta	2001	15	Borderline	5-FU	60	NA	NA/30/8	100
Massuco	2006	28	Borderline Unresectable	GemOx	39	38	15/21/10	87
Small	2008	39	Resectable Borderline Unresectable	Gem/XRT	33	NA	76% at 1 year	94
Katz	2008	160	Borderline	Gem/XRT	41	27	NA/40/13	94
McClaine	2010	29	Borderline	Gem/XRT	41	42	NA/23.3/15.5	67
Patel	2011	17	Borderline	Gem/Tax Cape 5-FU/XRT	64	22	15/NA/NA	89
Stokes	2011	40	Borderline	Cape/XRT	40	58	12/23/NA	88
Takahashi	2013	80	Borderline	Gem/XRT/LP	51	NR	34% at 5 years	100
Christians	2014	18	Borderline	FOLFIRINOX Gem/XRT Cape/XRT	67	83%	NA/NA/9.3	100
Rose	2014	64	Borderline	Gem/Tax	48	48	23.6/NA/15.4	87
Blazer	2015	43	Borderline Unresectable	FOLFIRINOX GemOx/XRT	51	18	21.2/NA/12.7	86

Pts—Patients; Chemo—Chemotherapy; R—Resected cohort; UR—Unresected cohort; Gem—Gemcitabine; Ox—Oxaliplatin; Tax—Taxane; Cape—Capecitabine; XRT—External radiation therapy; NA—Not available.
